# DNA Damage Repair Profiles Alteration Characterize a Hepatocellular Carcinoma Subtype With Unique Molecular and Clinicopathologic Features

**DOI:** 10.3389/fimmu.2021.715460

**Published:** 2021-08-12

**Authors:** Peng Lin, Rui-zhi Gao, Rong Wen, Yun He, Hong Yang

**Affiliations:** Department of Medical Ultrasound, The First Affiliated Hospital of Guangxi Medical University, Nanning, China

**Keywords:** hepatocellular carcinoma, DNA damage repair, multi-omics, immune, survival

## Abstract

Hepatocellular carcinoma (HCC) is one of the most common malignancies and displays high heterogeneity of molecular phenotypes. We investigated DNA damage repair (DDR) alterations in HCC by integrating multi-omics data. HCC patients were classified into two heterogeneous subtypes with distinct clinical and molecular features: the DDR-activated subtype and the DDR-suppressed subtype. The DDR-activated subgroup is characterized by inferior prognosis and clinicopathological features that result in aggressive clinical behavior. Tumors of the DDR-suppressed class, which have distinct clinical and molecular characteristics, tend to have superior survival. A DDR subtype signature was ultimately generated to enable HCC DDR classification, and the results were confirmed by using multi-layer date cohorts. Furthermore, immune profiles and immunotherapy responses are also different between the two DDR subtypes. Altogether, this study illustrates the DDR heterogeneity of HCCs and is helpful to the understanding of personalized clinicopathological and molecular mechanisms responsible for unique tumor DDR profiles.

## Introduction

Liver cancer is the sixth most common cancer and the third most frequent cause of cancer-related death globally (1). Hepatocellular carcinoma (HCC), the most common form of liver cancer, accounts for about 90% of all cases and frequently develops in patients who are infected by hepatitis B virus (HBV) or hepatitis C virus (HCV), alcohol abuse, or metabolic syndrome (2). HCC commonly leads to inferior survival and requires molecules that help in refining prognosis and monitoring treatment response. Any attempt to improve the prognosis of HCC should involve clear recognition of HCC molecular characteristics. To date, several studies have proposed molecular and immune classifications of HCC based on genomic, transcriptomic, and proteomic data (3–5). These subtyping strategies broaden the knowledge into the molecular phenotype of HCC and provide effective targeted therapy options. However, the molecular mechanisms’ response for the dismal prognosis of HCC are still unclear.

DNA damage repair (DDR) genes are the key to maintaining the stability of the human genome. Conversely, the loss of DDR function could lead to the onset and progression of cancer (6). Furthermore, treatment strategies focused on altered DDR function are becoming gradually realized. For example, Poly (ADP-ribose) polymerase (PARP), nuclear enzymes that recognize DNA damage, have been a therapeutic target for cancer treatment (7). DDR genes could be divided into some functional pathways based on their specific function in relation to DNA damage (8). Previously, The Cancer Genome Atlas (TCGA) work group comprehensively analyzed the influences of DDR pathway-related genes in cancers (8). The excellent study provides a rich resource for mechanistic and therapeutic analysis of cancer. However, transcriptomic and proteomic analysis of HCC from the perspective of DDR gene dysregulation and heterogeneity is still limited, especially in HCC. HCCs are complex ecosystems characterized by heterogeneity of molecular features and immune infiltrations. DDR actively participated in the processes of HCC carcinogenesis and immune characteristics. Recently, Yang et al. found that an important DDR gene TP53, its neoantigen may influence survival of HCC patients by regulating anti-tumor immunity thus could be an effective immunotherapy biomarker (9). Xu et al. also explored relationships between DDR gene RAD51 and immune infiltration in HCC (10). However, these studies mainly focused on role of single DDR gene in immune characteristics of HCC. Therefore, it is imperative to uncover the roles of DDR in HCC.

Here, we aim to comprehensively analyze transcriptional profile alteration of DDR genes in HCC. We have successfully identified two DDR gene-based subtypes based on 276 DDR genes. The two DDR-based subtypes have distinct clinical outcomes and molecular characteristics. Our data based on pan-cancer analysis also reveals heterogeneity among different cancer types and provides an alternative immune treatment response prediction approach. Our data shed light on the aspects of DDR alterations in HCC, which could be useful in guiding immunotherapy and prognosis monitoring.

## Methods

### DNA Damage Repair Genes Curation

A total of 276 DDR genes were acquired from previous work by TCGA DDR-AWG (8, 11, 12). These genes were assembled based on MSigDB v5.0 and knowledge-based curation of DDR pathways. DDR genes mainly belong to ten DDR pathways: (1) base excision repair (BER); (2) nucleotide excision repair (NER); (3) mismatch repair (MMR); (4) the Fanconi anemia (FA) pathway; (5) homology-dependent recombination (HR); (6) non-homologous DNA end joining (NHEJ); (7) direct damage reversal/repair (DR); (8) translesion DNA synthesis (TLS); (9) nucleotide pool maintenance (NP); and (10) genes are either correlated with more than one DDR pathway, or coordinate cellular and molecular responses to DNA damage. This study of deidentified data was approved by the institutional review board of First affiliated hospital of Guangxi Medical University [2020(KY-E-119)].

### DNA Damage Repair Genes-Based Clustering

First, we evaluated the global DDR alteration and proposed DDR gene-based subtypes based on two HCC cohorts included in the study. (1) Training cohort: Considering TCGA includes multi-omics resources for analysis, we characterized DDR characteristics based on TCGA. 371 primary HCC patients with RNA-seq date and corresponding survival information available from TCGA-Liver Hepatocellular Carcinoma (TCGA-LIHC) dataset. The RNA-seq dataset and the corresponding clinical parameters were downloaded from UCSC-Xena (https://xenabrowser.net/datapages/). Gene expression value was transformed into log2 [Fragments Per Kilobase of transcript per Million mapped reads (FPKM) +1] for further analysis. (2) Validation cohort: 231 primary HCC RNA-seq and clinical information were downloaded from the International Cancer Genome Consortium (ICGC) dataset [accession ID: Liver Cancer RIKEN Japan (LIRI-JP)] dataset (13). Gene expression profiles were also converted into log2 (normalized read count + 1) for further analysis.

We performed K-means consensus clustering with transcriptomic profile of 276 DDR genes to identify subgroups. Consensus clustering was processed using the CancerSubtypes package in R software (14). The following details were set for subgrouping: number of repetitions = 1,000 bootstraps; pItem = 0.8 (resampling 80% of any sample); maxK=6 (k-means clustering with up to 6 clusters). An appropriate number of clusters was determined based on the clustering results and clinical ease of use. Similar clustering processes were performed in the training and validation cohorts. The Kaplan-Meier (K-M) method with log-rank test was performed to compare overall survival (OS) differences between the two subgroups.

### Clinical and Molecular Characteristics Specific for the DDR Subtype

To observe clinicopathological and molecular characteristics between different DDR subtypes. We also compared clinicopathologic and molecular features between the two subgroups. Chi-square test was used to explore clinicopathological feature distribution between different DDR subtypes. The somatic mutation profile of HCC patients from TCGA was also downloaded from the TCGA database and ICGC, respectively. The somatic mutation data were further analyzed using the “maftools” R package (15).

We also compared transcriptomic alterations between the DDR-activated subtype and the DDR-suppressed subtype by using gene set enrichment analysis (GSEA). The GSEA procedures were performed based on the ClusterProfiler package in R software (16).

Here, we further conducted a metagene approach proposed previously for 28 immune cell subpopulations for HCC tumor microenvironment evaluation (17). Using the gene set variation analysis (GSVA) algorithm, the relative infiltration score of 28 immune cell subpopulations was estimated (18). Metagenes for 28 immune cell subpopulations were obtained from a previous study (17). Then, immune profile differences between subtypes were estimated by Wilcoxon test.

### DDR Subtype Signature Development and Validation

Considering too many genes, detection is hard for clinical application. We developed a gene signature for DDR subtype identification. Differentially expressed genes between DDR-activated and DDR-suppressed subtypes were identified by using Wilcoxon analysis. DDR genes with log2 (fold change)>1 and P-value <0.05 were considered as DDR subtype specific genes. In the era of precision medicine, proteogenomics could provide information about more direct executors and thus help in making a more precise diagnosis and prognosis monitoring of cancers. Considering that DDR-related proteins were major executors, we further compared the relationships between transcriptomic level and proteomic level. Proteomics data of 159 HCC patients were required from the clinical proteomic tumor analysis consortium (CPTAC) data portal (3). In the CPTAC cohort, 10,783 quantified protein expression levels were identified based on the Isobaric tandem mass tags (TMT) approach. Pairing transcriptomic and proteomic data were identified by Spearman correlation analysis. Genes that showed a significant correlation (spearman correlation coefficient >0.4) between protein levels and mRNA levels were submitted to DDR subtype signature construction. DDR genes that were significantly up-regulated in the DDR-activated subgroup and had high correlations between protein and mRNA levels were used for DDR subtype signature development. The DDR subtype signature score was calculated based on the average expression of the included DDR genes.

### Prognostic Value of DDR Subtype Signature

To test the performance of the DDR subtype signature in survival prediction, five cohorts of HCC patients were included, including two RNA-seq datasets (TCGA, ICGC), two gene chips datasets acquired from Gene Expression Omnibus (GEO) [accession number: GSE14520 (19) and GSE54236 (20)] and proteomics dataset CPTAC (3). GSE14520 includes 242 HCC patients, while GSE54236 includes 78 HCC patients. Subsequently, we also explore whether the DDR-subtype signature could be a pan-cancer survival indicator. Therefore, RNA-seq data of 7779 cancer patients from 20 types of cancer were also downloaded from the TCGA database similar to the TCGA HCC download pipeline. Univariate Cox analyses were conducted in each cancer type to explore relationships between DDR subtype signature and OS. Hazard ratio (HR) and corresponding 95% corresponding interval (CI) were calculated. Then, Stata 14.0 software was used to integrate survival analysis results. Heterogeneity analyses used the I^2^ and Q tests. When I^2^>50% and the Q test *P* <0.1, it was considered that there was heterogeneity, and the random effect model was selected.

### DDR Subtype Signature for Immunotherapy Response Prediction

To validate the value of the DDR subtype signature in immunotherapy prediction, we analyzed relationships between the DDR signature and immunotherapy response from the IMvigor210 cohort (21). The IMvigor210 cohort included 348 patients with locally advanced or metastatic urothelial cancer treated with an anti-PD-L1 agent (atezolizumab). The Kruskal-Wallis test was used to explore DDR signature score differences among different immunotherapy response groups [complete response (CR), partial response (PR), stable disease (SD), progressive disease (PD)]. The area under curve (AUC) was used to estimate the DDR signature for immunotherapy response (CR/PR VS. SD/PD).

### Single-Cell Analysis for DDR Heterogeneity Estimation

Single-cell data could provide higher resolution of gene alteration information. After filter out low low-quality cells, single-cell transcriptomic data of 12162 cells from 12 primary HCC samples was used for analysis from previous study (22). To explore DDR signature heterogeneity in different cell types, we calculated DDR signature in each cell and compared difference among different cell types. Seurat R package was used to generate t-SNE plot for cell types visualization.

## Results

### DDR Gene Alteration Profiles in HCCs

**Figure 1:**
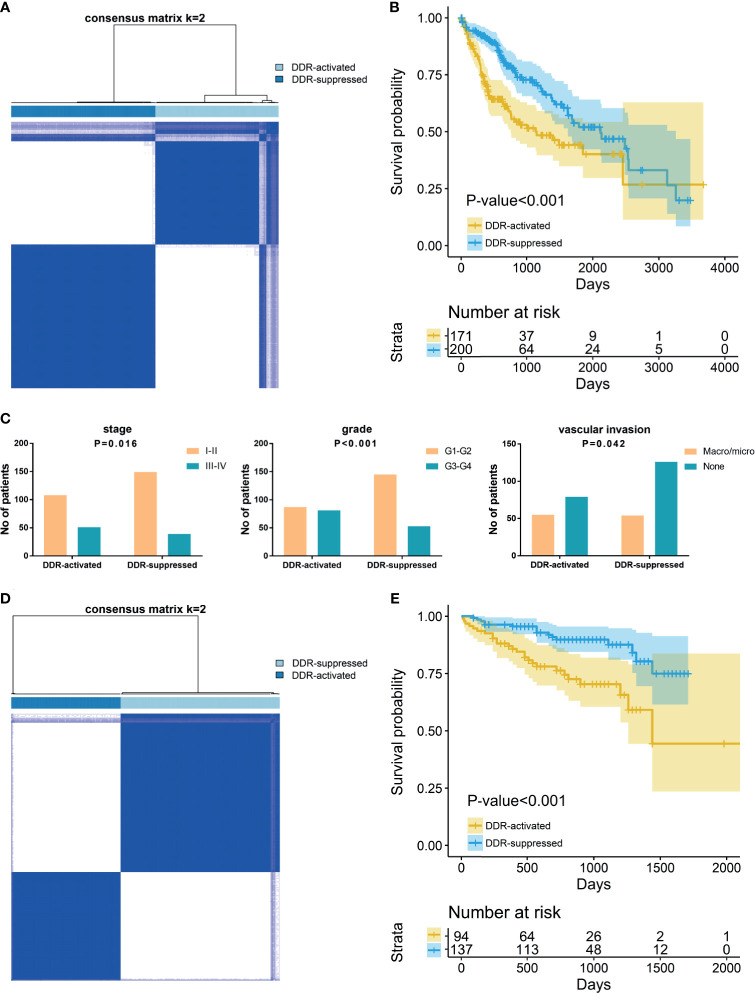
Consensus clustering for DNA damage repair (DDR) related genes in HCC patients. **(A)** The consensus matrix shows patients with two distinct DDR statuses in the TCGA dataset. **(B)** Kaplan-Meier curves for overall survival based on DDR subgroups (Log-rank test) in TCGA dataset; **(C)** Tumor stage, grade, and vascular invasion distribution differences between DDR subgroups; **(D)** The consensus matrix shows patients with two distinct DDR statuses in the ICGC dataset; **(E)** Kaplan-Meier curves for overall survival based on DDR subgroups (Log-rank test) in ICGC dataset.

To reveal the DDR gene heterogeneity of HCCs, all 371 HCC patients were divided into heterogeneous subtypes based on 276 DDR gene expression profiles (**Figure 1**). Considering the consensus clustering results and clinical significance, two DDR subgroups were identified. Cluster 1 (n=171, 46.1% of all HCCs) was designated as the DDR-activated subtype, owing to the relative upregulation of most DDR-related genes in this cluster. Cluster 2 (n=200, 53.9% of all HCCs), thereafter designated as the DDR-suppressed subtype based on the relative downregulation of DDR genes (**Figure 1A**). Furthermore, the two subtypes showed distinct clinical outcomes. K-M plots suggested that patients who were divided into DDR-activated subgroups suffered inferior OS (**Figure 1B**). We also compared clinical parameters between the two groups and found that advanced stage (chi-square value =5.757, P=0.016), high grade (chi-square value =18.013, P<0.001), and presence of vascular invasion (chi-square value = 4.135, P=0.042) were more frequently observed in the DDR-activated subgroup (**Figure 1C**).

In the validation ICGC cohort, all 231 HCCs were also divided into different subtypes based on the 276 DDR gene expressions. Similarly, K-means clustering indicated that patients who were also categorized into two subgroups had similar DDR pathway alterations with the training cohort (**Figure 1D**). Patients were also divided into DDR-activated and DDR-suppressed subgroups. A similar survival difference between two subgroups was also observed (**Figure 1E**). These findings further validate the inferior prognosis of patients in the DDR-activated group.

### DDR Genes-Based Subtypes Show Distinct Clinical and Molecular Characteristics

**Figure 2:**
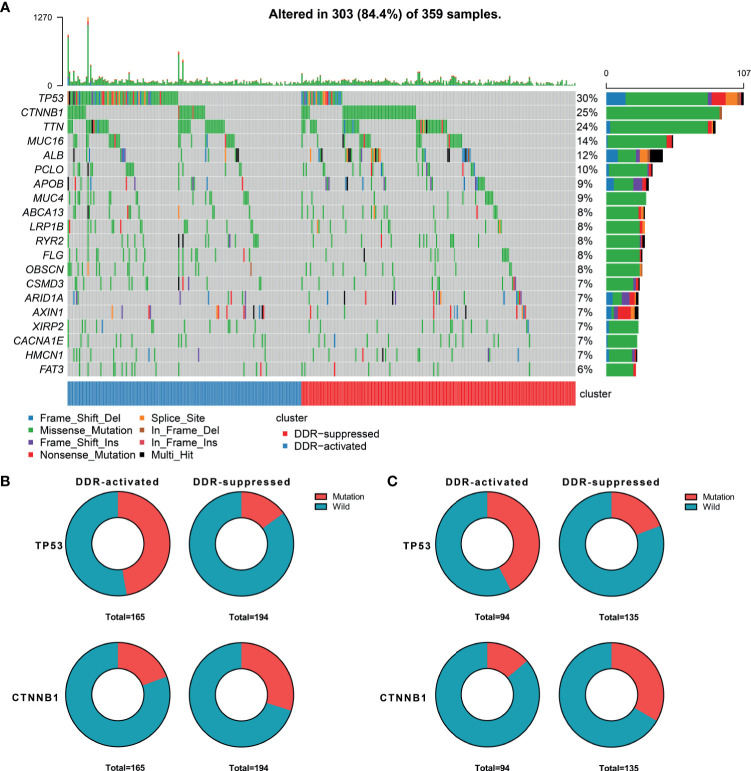
Genomic alterations between DDR-activated and DDR-suppressed subgroups. **(A)** Landscape of mutation profiles in HCC samples. Mutation information of each gene in each sample is shown in the waterfall plot. Top panel shows individual tumor mutation burden. The data shown were analyzed based on the TCGA data portal. **(B)** The mutation rate of TP53 was higher in the DDR-activated subgroup, while CTNNB1 was higher in the DDR-suppressed subgroup in the TCGA dataset. **(C)** The mutation rate of TP53 was higher in the DDR-activated subgroup, while CTNNB1 was higher in the DDR-suppressed subgroup in the ICGC dataset.

When considering genomic alterations, we also compared gene mutation differences between two DDR subtypes. The most common mutational genes in patients from the training cohort were TP53 and CTNNB1 (**Figure 2A**). Considering the importance of these two genes, we compared and found that TP53 was more frequently mutated in the DDR-activated subgroup (78/165 Vs. 29/194, chi-square= 44.53, P<0.001) while CTNNB1 was more frequently mutated in the DDR-suppressed subgroup (32/165 Vs. 58/194, chi-square= 5.24, P=0.022, **Figure 2B**). In the validation cohort, we found TP53 also frequently mutated in the DDR-activated subgroup (40/94 Vs. 26/135, chi-square= 14.66, P<0.001) while CTNNB1 was more frequently mutated in the DDR-suppressed subgroup (13/94 Vs. 45/135, chi-square= 11.15, P=0.001, **Figure 2C**).

**Figure 3:**
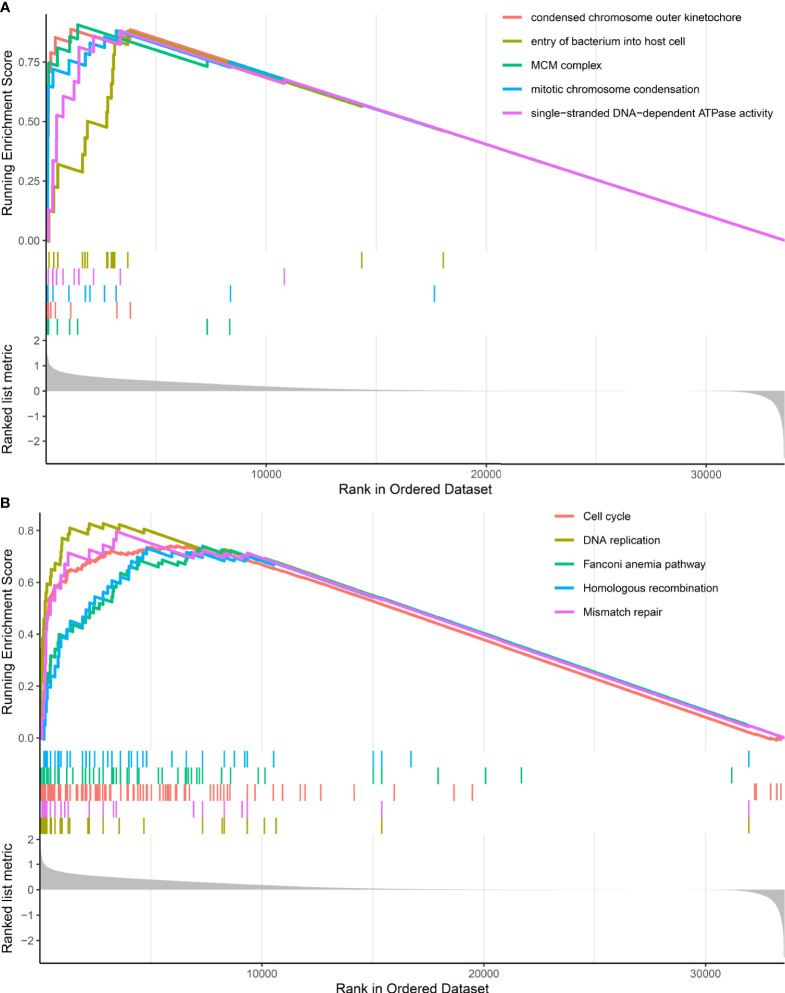
Gene set enrichment analysis of DDR-subtype specific pathway analysis. **(A)** Top five most significant altered gene ontology terms in the DDR-activated subgroup when compared with the DDR-suppressed subgroup. **(B)** Top five most significant altered KEGG pathways in the DDR-activated subgroup when compared with the DDR-suppressed subgroup.

GSEA analysis revealed that DDR subtypes have distinct transcriptomic alterations. The top five most activated gene ontology terms in the DDR-activated subgroup were MCM complex, condensed chromosome outer kinetochore, mitotic chromosome condensation, single-stranded DNA-dependent ATPase activity, and entry of the bacterium into the host cell (**Figure 3A**). The top five most activated Kyoto Encyclopedia of Genes and Genomes terms in DDR-activated subgroup were DNA replication, mismatch repair, cell cycle, Fanconi anemia pathway, and homologous recombination (**Figure 3B**).

### DDR Subtypes Characterized Different Immune Profiles

**Figure 4:**
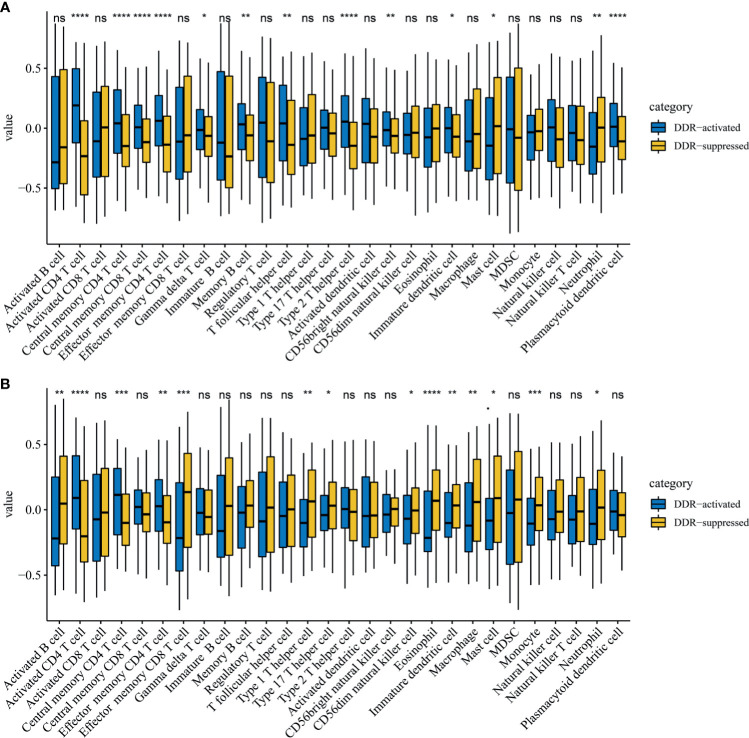
Immune profile alterations between the DDR-activated and DDR-suppressed subgroups. **(A)** TCGA; **(B)** ICGC. * represents P < 0.05, ** represents P < 0.01,*** represents P < 0.01, **** represents P < 0.0001, ns represents no significant difference.

Immune cell infiltration markedly influenced tumor progression and immunotherapy treatment response. Therefore, we also explored differences in immune cell infiltrations between two DDR subtypes. Notably, activated CD4 T cells, central memory CD4 T cells, and effector memory CD4 T cells were significantly up-regulated in the DDR-activated subgroup regardless of the training (P=2.39E-17, 1.83E-06 and 7.01E-09 respectively, **Figure 4A**) and validation cohort (P=8.78E-07, 5.52E-04 and 1.70E-03 respectively, **Figure 4B**). Mast cell and neutrophil cell were significantly up-regulated in DDR-suppressed subgroup in the training (P=0.025 and 0.001 respectively, **Figure 4A**) and validation cohort (P=0.017 and 0.014 respectively, **Figure 4B**).

### DDR Subtype Signature Is a Prognostic Indicator for HCC’s OS

**Figure 5:**
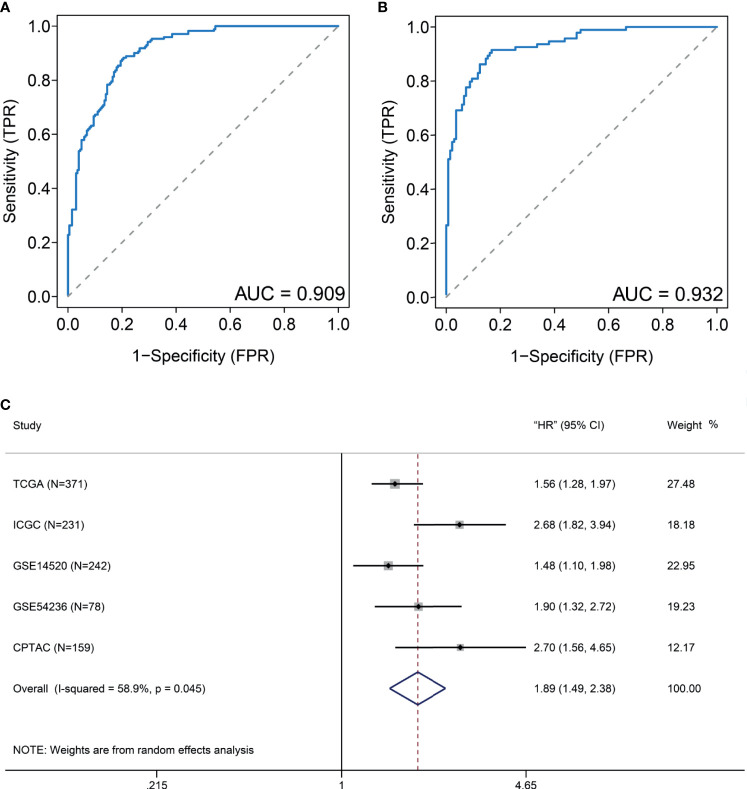
DDR-subtype development and validation. Receiver operating characteristic curve (ROC) analyses of DDR signature to evaluate its performance in TCGA **(A)** and ICGC **(B)** datasets. **(C)** Forest plots show that a high DDR signature score is correlated with inferior overall survival based on five cohorts.

Considering that many gene expression detections are difficult for clinical implication, it is imperative to have a signature that could be used for DDR subtype identification. Differential analysis indicated that 11 DDR-related genes, including TYMS, RRM2, UBE2T, HMGB2, SOX4, FEN1, RFC4, H2AFX, FANCI, PCNA, and RMI2, were most specifically upregulated in the DDR-activated subtype. Correlation analyses from the CPTAC cohort found that six genes (FEN1, H2AFX, HMGB2, PCNA, RFC4, and RRM2) were significant correlated between transcriptomic and proteomic data. Therefore, we used the average expression of six markers for the DDR-activated signature. AUC of ROC indicated that the gene signature could be useful for stratification patients in different DDR subtypes (AUC= 0.909 in training cohort, **Figure 5A**; AUC= 0.932 in validation cohort, **Figure 5B**). Therefore, the six DDR gene signatures provided an alternative and clinically accessible method for DDR subtype identification.

**Figure 6:**
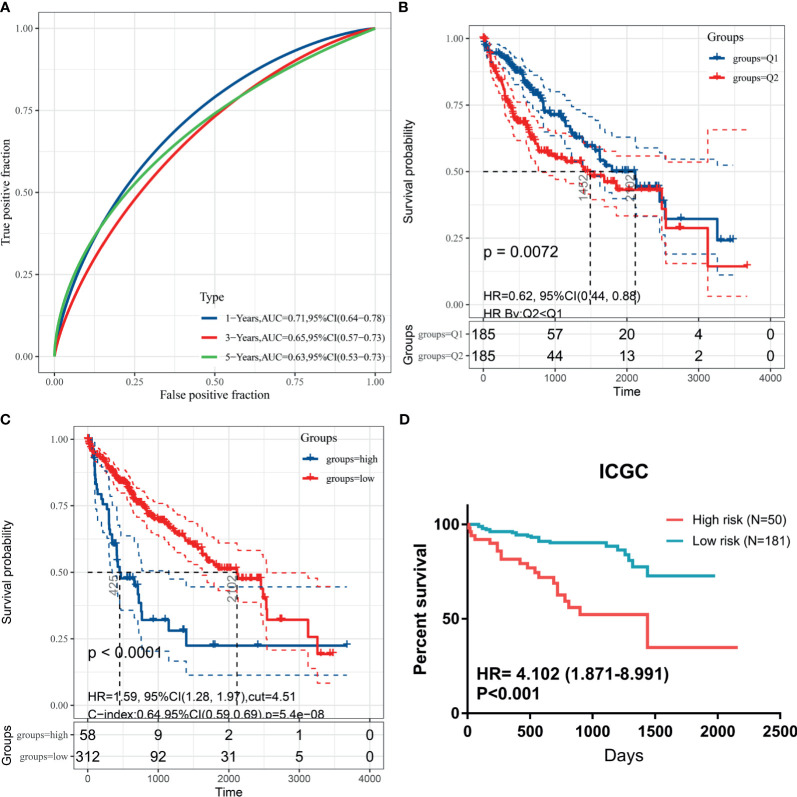
Kaplan-Meier plots for DDR signature cut-off identification. **(A)** Time-dependent ROC for DDR signature survival prediction in TCGA database; **(B)** survival difference between high and low DDR signature based on median value; **(C)** survival difference between high and low DDR signature based on best separation in training cohort; **(D)** cut-off from TCGA cohort could be useful for risk stratification in ICGC dataset.

To validate generalization performance of DDR subtype signature in different cohorts, a meta-analysis approach was utilized to integrate survival analysis results from five cohorts (TCGA, ICGC, GSE14520, GSE54236, and CPTAC). The DDR-subtype signature showed statistical significance in five cohorts. Meta-analysis revealed that a higher signature showed inferior OS (HR, 1.89; 95% CI, 1.49–2.38, **Figure 5C**). Time-dependent ROC was generated and showed that area under curves for 1, 3, 5 years were 0.71, 0.65 and 0.63 respectively (**Figure 6A**). K-M plot showed that patients could be divided into two groups with distinct prognosis based on median value of DDR signature (**Figure 6B**). To find optimal cut-off of DDR gene signature for risk stratification, we also evaluated the best significant cut-off value (**Figure 6C**). The optimal cut-off value was 4.51, which was also effective for risk stratification in ICGC cohort (**Figure 6D**).

**Figure 7:**
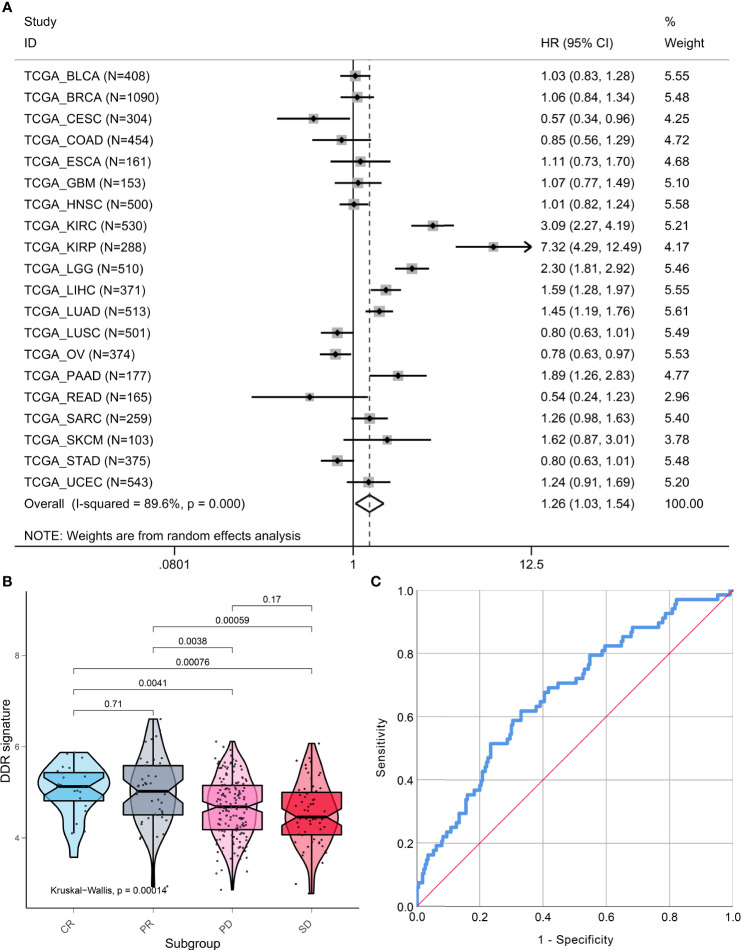
Pan-cancer prognostic value and immunotherapy response prediction of DDR signature. **(A)** Pan-cancer analysis to evaluate prognostic value of DDR signature. **(B)** TME scores in groups with different anti-PD-L1 clinical response statuses. **(C)** A receiver-operating characteristic (ROC) curve was used to measure the performance of the DDR subtype signature in immunotherapy response prediction.

Pan-cancer analysis that included 7779 patients from 20 types of cancer indicated that the DDR signature still remains a prognostic indicator. A higher DDR signature score suggested that patients had poor survival (HR, 1.26; 95% CI, 1.03, 1.54; **Figure 7A**). However, marked heterogeneity was observed among different cancer types (I-squared = 89.6%, p <0.001). For example, two pathological subtypes of lung cancer, lung adenocarcinoma and lung squamous cell cancer, showed distinct prognoses of the DDR-subtype signature.

### DDR Signature Is a Promising Predictor for Immunotherapy

To explore the DDR subtype signature for immunotherapy response prediction, we explored 348 samples from the IMvigor210 cohort. For gene expression analyses with respect to response, 298 patients were used to estimate DDR subtype score for immunotherapy response prediction. We found that the DDR signature score was higher in the CR or PR group when compared with the SD and PD groups (Kruskal-Walls, P = 0.00014; **Figure 7B**). The results of the ROC curve indicated that the DDR signature could be used for immunotherapy response prediction (AUC=0.671, 95% CI, 0.598-0.743, P<0.001; **Figure 7C**).

### DDR Signature Is Heterogeneous in Tumor Immune Microenvironment

**Figure 8:**
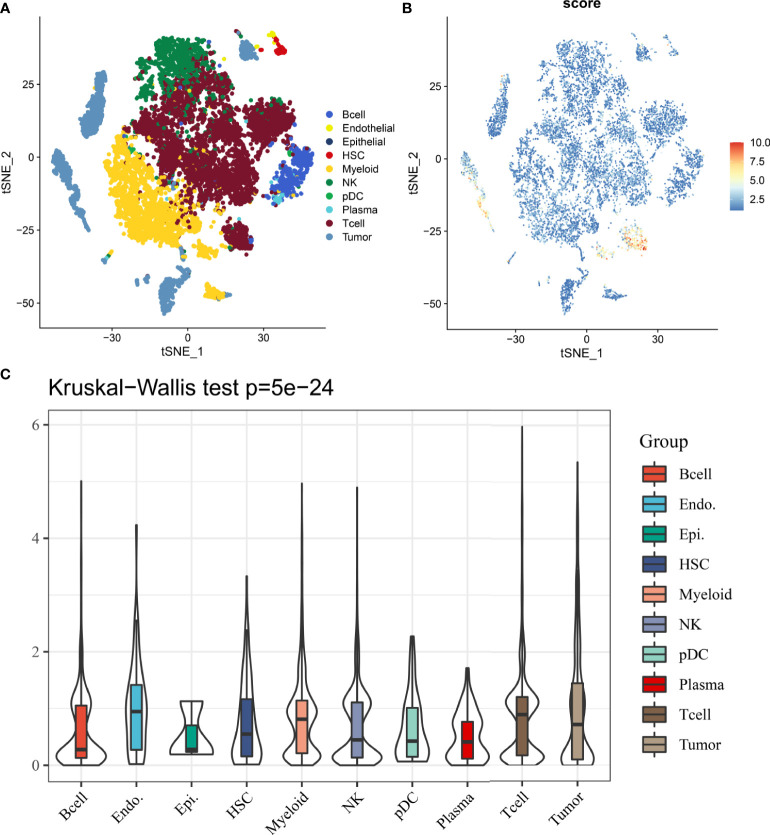
The distribution and expression of DDR subtype signature in HCC. **(A)** The percentage of each type of cells in HCC. **(B)** The distribution of each type and DDR score expression in HCC. **(C)** DDR scores in different cells are various.

In 12162 cells from 12 samples, cells were mainly divided into 10 types, including B cell, endothelial, epithelial, hepatic stellate cells (HSCs), myeloid, NK, pDC, plasma, T cell and tumor cell (**Figure 8A**). Results from single-cell analysis found that DDR signature score was significant different distribute in different clusters (**Figure 8B**). And DDR score was significant up-regulated in some particular clusters, especially for tumor and T cells. Kruskal-Wallis test also showed that DDR score was significant different among different cells (**Figure 8C**).

## Discussion

Despite the progress in the approaches to therapy, the prognosis of HCC remains poor owing to the high recurrence rate, even after surgical resection. Molecular heterogeneity often tends to limited treatment options and is a challenge for survival monitoring. Hence, some excellent previous studies that aimed at molecular-phenotypic subtype identification of HCC have provided novel insights into HCC precision medicine (3, 4). However, the roles of DDR in the ecosystems of HCC still need to be deciphered. In this study, we analyzed multi-omics data that included genomics, transcriptomics, and proteomics to characterize differences between DDR-based subtypes in HCC. Further study also explored immunotherapy response and immune profile differences between DDR-based subtypes.

Our integrated analysis revealed that HCC patients have two distinct DDR statuses: the DDR-activated subtype and the DDR-suppressed subtype. Patients in the DDR-activated subgroup are characterized by aggressive clinical behavior, including advanced stage, poor differentiation, and inferior prognosis. To identify the molecular characteristics of distinct DDR subtypes in HCC, we found that genomic alterations were significant between the two subtypes. TP53 mutation was more frequently observed in the DDR-activated subtype. The tumor suppressor p53 plays a key role in DNA repair and somatically mutated in many types of human cancers, including HCC (23). As the “guardian of the genome,” TP53 mutations have been clinically recognized as an inferior survival indicator for HCC (24). Interestingly, in the DDR-suppressed subgroup, CTNNB1 was more frequently mutated when compared with the DDR-activated subgroup. CTNNB1 mutations activating ß-catenin and were mutually exclusive with TP53 (25). A previous excellent proteogenomics study revealed that the protein and phosphorylation differences between CTNNB1 mutant and wild-type HCC were mainly concentrated in metabolic pathways (3). In the era of immunotherapy, more studies have found that Wnt/CTNNB1 mutations are the characterization of immune-excluded class HCC (26, 27). Harding et al. showed that HCC patients with CTNNB1 mutations did not respond to PD-1 blocking therapy, which validated the hypothesis that HCC “cold tumors” defined by Wnt/CTNNB1 mutations are not responsive to immunotherapy (28).

GSEA analysis further indicated that our subgroup plan is credible. The DDR opens news perspectives for understanding the regulatory mechanisms of tumors. We also explore the immune microenvironment in HCCs. DDR subtypes have distinct immune profiles. Activated CD4 T cells, central memory CD4 T cells, and effector memory CD4 T cells were significantly up-regulated in the DDR-activated subgroup. CD4+ T cells can target tumor cells in a variety of ways, either by eliminating tumor cells directly through cytolytic mechanisms or indirectly by regulating TME (29, 30). Mast cell and neutrophil cell were specifically enriched in the DDR-suppressed subgroup. Tumor-infiltrating mast cells have been identified as being associated with resistance to anti-PD-1 therapy (31). These findings have shown that DDR subtypes have distinct immune cell infiltration differences, which hints at different immunotherapy responses between subtypes. Therefore, we also found immunotherapy response differences between distinct DDR subtypes. By applying ROC curve analysis, we also identified that the DDR subtype signature is valuable for immunotherapy response in patients with metastatic urothelial cancer treated with the anti–PD-L1 agent. We found that the DDR subtype signature was significantly higher in responders than in non-responders undergoing checkpoint blockade therapy. However, the performance of this signature in HCC should be further tested through analysis of a large cohort of HCC patients who have received immunotherapy.

To speed up clinical use, six DDR genes were composed as a signature for DDR-subtype identification. The signature showed high performance in dividing patients into distinct DDR subtypes in the training and validation cohort. The combination of RNA-seq data and mass spectrometry-based proteomics could provide a more comprehensive view globally. The DDR signature we proposed showed moderate prognostic value in HCC patients based on RNA-seq, microarray, and proteomics data. Their results also hinted that our results are robust and repeatable. However, pan-cancer analysis suggested that the prognostic value of the DDR signature is its heterogeneity. DDR alterations characteristics in different cancer types should be further analyzed.

Our study is not without its limitations. First, there is a lack of randomized trials of HCC patients who receive immunotherapy to validate the immunotherapy response prediction performance of the signature. Second, different expression detection platforms were used in our study, including RNA-seq, gene chip, and proteomics. Future studies are needed to validate the optimal cut-off for DDR subtype identification. Third, our study mainly focused on multi-cohort data for providing solid information for DDR-related survival information and molecular characteristics. Future *in vivo* and/or *in vitro* mechanism exploration may provide more information for DDR subtype alterations.

In conclusion, this study provides evidence of DDR heterogeneity and DDR categorized subtypes in HCC patients. Specific DDR subtype characteristics provide information for HCC clinical management and decision-making assistance. Our DDR subtype signature facilitates a deeper understanding of the mechanisms associated with HCC inferior prognosis and assists in developing more effective therapeutic targets and biomarkers for immunotherapies in HCC patients.

## Data Availability Statement

Data generated and analyzed during the current study are available from the UCSC TCGA data portal (http://xena.ucsc.edu/public/), ICGC (dataset ID: LIRI-JP, https://icgc.org/), CPTAC (https://proteomics.cancer.gov/programs/cptac) and GEO databases (dataset ID: GSE14520 and GSE54236, https://www.ncbi.nlm.nih.gov/).

## Ethics Statement

The studies involving human participants were reviewed and approved by First Affiliated Hospital of Guangxi Medical University. Written informed consent for participation was not required for this study in accordance with the national legislation and the institutional requirements.

## Author Contributions

Conceptualization: PL and R-zG. Methodology: PL, R-zG, and RW. Validation: YH and HY. Formal analysis: PL, R-zG, and RW. Data curation: YH and HY. Writing—original draft preparation: PL, R-zG, and RW. Writing—review and editing: YH and HY. Visualization: YH and HY. Supervision: YH and HY. All authors contributed to the article and approved the submitted version.

## Funding

This study has received funding by the Fund of National Natural Science Foundation of China (grant no. NSFC81860319).

## Conflict of Interest

The authors declare that the research was conducted in the absence of any commercial or financial relationships that could be construed as a potential conflict of interest.

## Publisher’s Note

All claims expressed in this article are solely those of the authors and do not necessarily represent those of their affiliated organizations, or those of the publisher, the editors and the reviewers. Any product that may be evaluated in this article, or claim that may be made by its manufacturer, is not guaranteed or endorsed by the publisher.
